# Magnesium-alloy rods reinforced bioglass bone cement composite scaffolds with cortical bone-matching mechanical properties and excellent osteoconductivity for load-bearing bone in vivo regeneration

**DOI:** 10.1038/s41598-020-75328-7

**Published:** 2020-10-23

**Authors:** Huyang Duan, Chuanliang Cao, Xiaolei Wang, Jun Tao, Chen Li, Hongbo Xin, Jing Yang, Yulin Song, Fanrong Ai

**Affiliations:** 1 Department of Orthopedic Surgery, The Second Affiliated Hospital of Nanchang University, Nanchang, 330006 Jiangxi China; 2 Department of Orthopedic Surgery, Yingtan People’s Hospital, Yingtan, 335000 Jiangxi China; 3 School of Mechanic and Electronic Engineering, Nanchang University, Nanchang, 330031 Jiangxi China; 4 Institute of Translational Medicine, Nanchang University, Nanchang, 330031 Jiangxi China; 5 School of Pharmacy, University of Nottingham, Nottingham, NG7 2RD UK

**Keywords:** Chemical modification, Metals, Chemistry, Engineering, Materials science

## Abstract

Various therapeutic platforms have been developed for repairing bone defects. However, scaffolds possess both cortical bone-matching mechanical properties and excellent osteoconductivity for load-bearing bone defects repair is still challenging in the clinic. In this study, inspired by the structure of the ferroconcrete, a high-strength bifunctional scaffold has been developed by combining surface-modified magnesium alloy as the internal load-bearing skeleton and bioglass-magnesium phosphate bone cement as the osteoconductive matrix. The scaffold combines the high mechanical strength and controllable biodegradability of surface-modified magnesium alloy with the excellent biocompatibility and osteoconductivity of bioglass-magnesium phosphate bone cement, thus providing support for load-bearing bone defects and subsequently bone regeneration. The scaffolds generate hydroxyapatite (HA) during the degrading in simulated body fluid (SBF), with the strength of the scaffold decreasing from 180 to 100 MPa in 6 weeks, which is still sufficient for load-bearing bone. Moreover, the scaffolds showed excellent osteoconductivity in vitro and in vivo. In a New Zealand White Rabbit radius defect model, the scaffolds degrade gradually and are replaced by highly matured new bone tissues, as assessed by image-based analyses (X-ray and Micro-CT) and histological analyses. The bone formation-related proteins such as BMP2, COL1a1 and OCN, all showed increased expression.

## Introduction

Large bone defects can result from a wide variety of causes, such as osteonecrosis, trauma, and cancer metastasis^1^. Although bone tissue has a remarkable ability to regenerate and heal itself, large bone defects which exceed the critical size cannot be fully and steadily repaired by themselves. Therefore, it’s necessary to graft autologous bone or artificial bone substitutes for treating the defects^2,3^. Although autologous bone grafting represents an effective approach for bone defects repairing, donor site morbidity and source-limitation have hampered its application in large bone defects. In contrast, artificial bone scaffolds have several distinct advantages such as abundant supply^4,5^.

Many bone substitutes based on single or composite materials have been fabricated for repairing large bone defects^6–9^. Metals are one of the desirable and wildly used biomaterials for load-bearing implants, attributing to their excellent mechanical properties and biocompatibility. Current clinically adopted metallic biomaterials include stainless steels, titanium and cobalt–chromium-based alloys^10^. Titanium alloys have been increasingly used in orthopedic surgeries due to their lower modulus, superior biocompatibility and enhanced corrosion resistance compared to stainless steels and cobalt-based alloys. However, the Young's moduli of the titanium alloys are still much higher than cortical bone, resulting in the stress shielding of surrounding bone, which has been identified as a major reason for implant loosening. Moreover, titanium alloys are non-absorbable in vivo, debris generated from frequent friction are almost inevitable after long-term usage (around 10 years)^11^. In comparison, magnesium-based alloys are lightweight and possess mechanical properties similar to cortical bone and degrade in the electrolytic environment of the body, which are desirable properties for load-bearing implants^12–14^. However, the rapid corrosion rate of magnesium in the electrolytic physiological environment is one of the greatest limitations for its use as orthopedic implants. In addition, the osteoconductivity of magnesium is unsatisfactory^15^.

Osteoconductivity is one of crucial characteristics for bone-regeneration scaffolds in the process of bone formation^6^. Bioglass is a biocompatible material with remarkable osteoconductivity and controllable biodegradability. However, its poor toughness restricts its clinical application for load-bearing defects^16–21^. Mechanical properties of an ideal bone scaffold should match host cortical bone and offer proper load transfer^22^.

Already numerous composites such as Mg-containing bioactive glasses was applied in areas such as bone cements, coatings, and scaffolds for bone tissue engineering^23^, results showed that it is possible to modulate some important scaffolds properties, such as crystallinity, degradation, and morphology, all of which are important for designing scaffolds which satisfy the requirements of specific engineered bone tissue. However, strength of these composites were not adequate for supporting bone mechanical loading^24^.

Therefore, for repairing load-bearing bone defects, it is of great clinical importance to develop a scaffold which possesses cortical-bone-matching mechanical property, controllable biodegradability and excellent osteoconductive property^25^. However, scaffolds that meet all above requirements have rarely been reported in the literature.

In the present work, inspired by the structure of the ferroconcrete, a novel scaffold has been fabricated by integrating surface-modified magnesium alloy rods with bioglass-magnesium phosphate bone cement, in which magnesium alloy rods are serving as the load-bearing component, while the bioglass-magnesium phosphate cement matrix is serving as the osteoconductive component. The composite scaffolds possessed mechanical properties matching cortical bone, excellent biocompatibility, controllable biodegradability and outstanding osteoconductivity which are all desirable properties for load-bearing bone defects repair and bone regeneration^15,18,26,27^. In order to improve the resistance of magnesium alloy to corrosion, the magnesium alloy rod surface was coated with polycaprolactone (PCL), allowing the degradation of the Mg alloy to match new bone formation. The cytocompatibility and osteoconductivity of the composite scaffolds were investigated in vitro and in vivo.

## Results

### Characterization of the scaffolds

**Figure 1:**
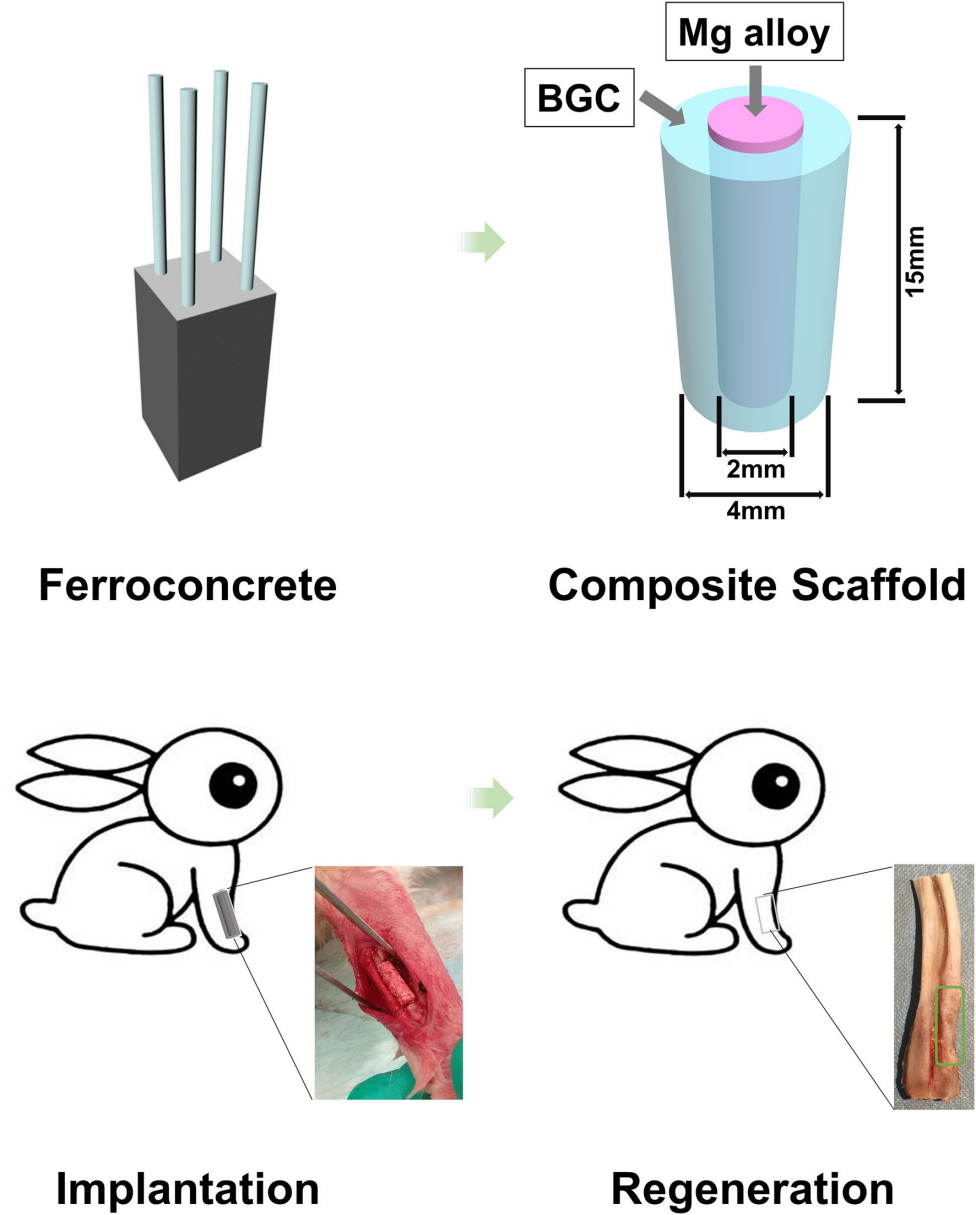
Schematic illustration of developing a high-strength bifunctional scaffold inspired by the structure of ferroconcrete structure, and the reparative process of rabbit radius bone defects followed by osteogenesis by the scaffold.

In order to improve the mechanical and osteoconductive properties of scaffolds for load-bearing bone defects repair, a high-strength bifunctional scaffold of surface-modified magnesium alloy reinforced bioglass-magnesium phosphate bone cement was constructed inspired from the structure of steel reinforced concrete architectures. As depicted in Fig. 1, PCL modified Magnesium (Mg) alloy (as shown in Supplementary Fig. S1) is similar with the inner steel of the reinforced concrete providing excellent mechanical properties. While the bioglass-magnesium phosphate bone cement is similar with the outer concrete providing the scaffold excellent osteoconductivity for load-bearing bone defects repairing.

**Figure 2:**
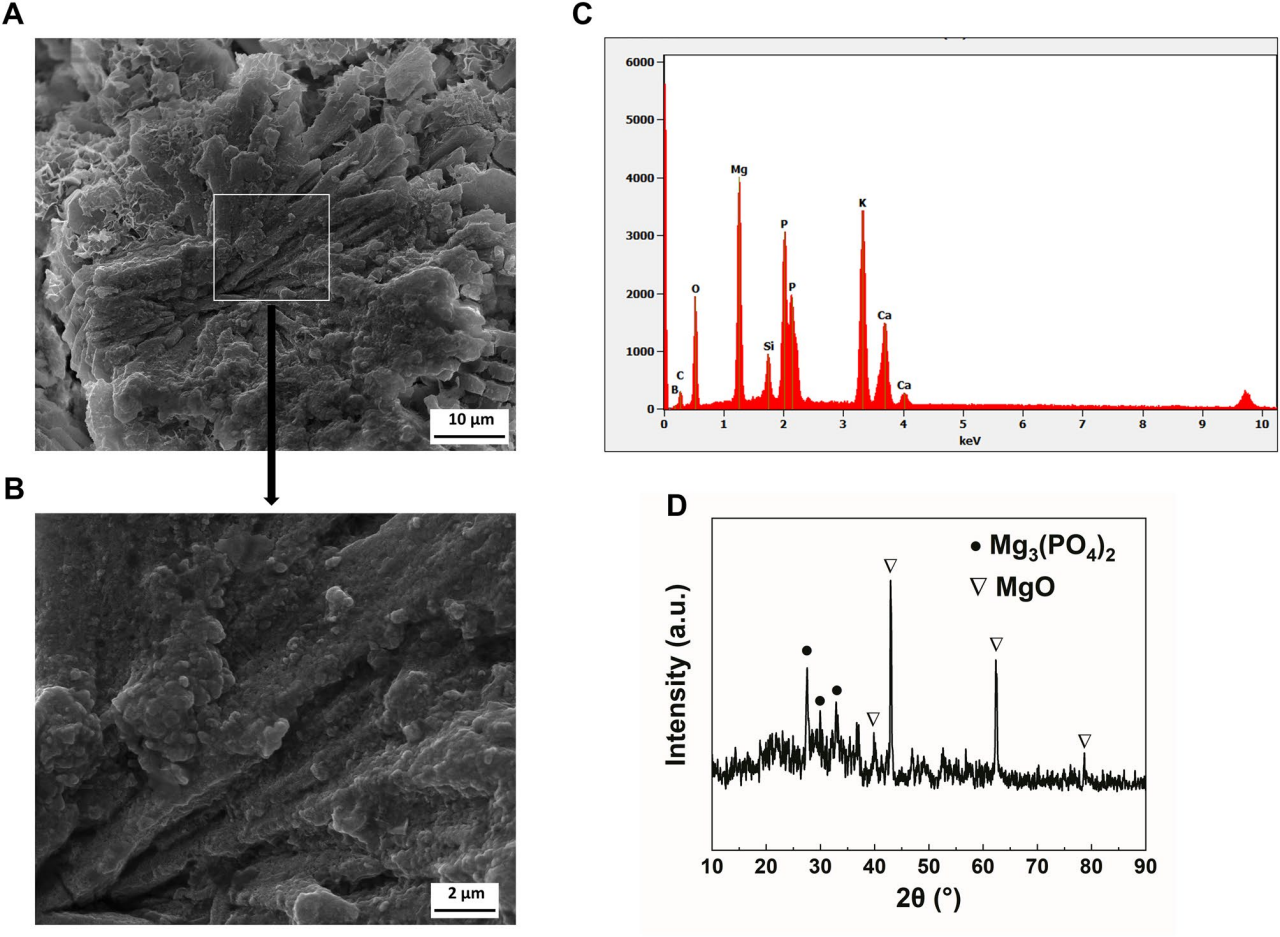
Characteristics of BGC. (**A**,**B**) SEM images of surface morphology, (**C**) Energy dispersive spectrum analysis data, (**D**) X-ray diffraction spectrum of BGC.

The surface microtopography of BGC were characterized by scanning electron microscopy (SEM), indicating that the BG particles were tightly bond with the magnesium phosphate cement (Fig. 2A,B). Columnar-like crystals were found in the sample represents magnesium phosphate which was formed via the reaction shown below:

1 $${\text{Mg}}^{2 + } + {\text{PO}}_{4}^{3 - } + 6{\text{H}}_{2} {\text{O}} = {\text{Mg}}_{3} \left( {{\text{PO}}_{4} } \right)_{2} \cdot 6{\text{H}}_{2} {\text{O}}$$

Due to the mineral-interaction between BGC particles and phosphates in the mixture, BG particles were uniformly dispersed in the magnesium phosphate cement matrix. Energy-dispersive X-ray spectroscopy (EDS) shows the distributions of Mg, P, Ca, Si, and B, indicating the homogeneous reaction of BG with MgP cement matrix as well (Fig. 2C). The crystal structure of BGC scaffolds was further characterized by X-ray diffractometry (XRD). As displayed in Fig. 2D, the BGC shows sharp characteristic peaks at 2θ = 43.2°, 62.7° and 37.1° that are attributed to MgO in consistency with JCPDS.75-1525, while peaks at 2θ = 27.6°, 32.9° and 29.9° are attributed to Mg_3_(PO_4_)_2_ in consistency with JCPDS.48-1167.

### Degradation behavior and mechanical properties of the scaffolds

**Figure 3:**
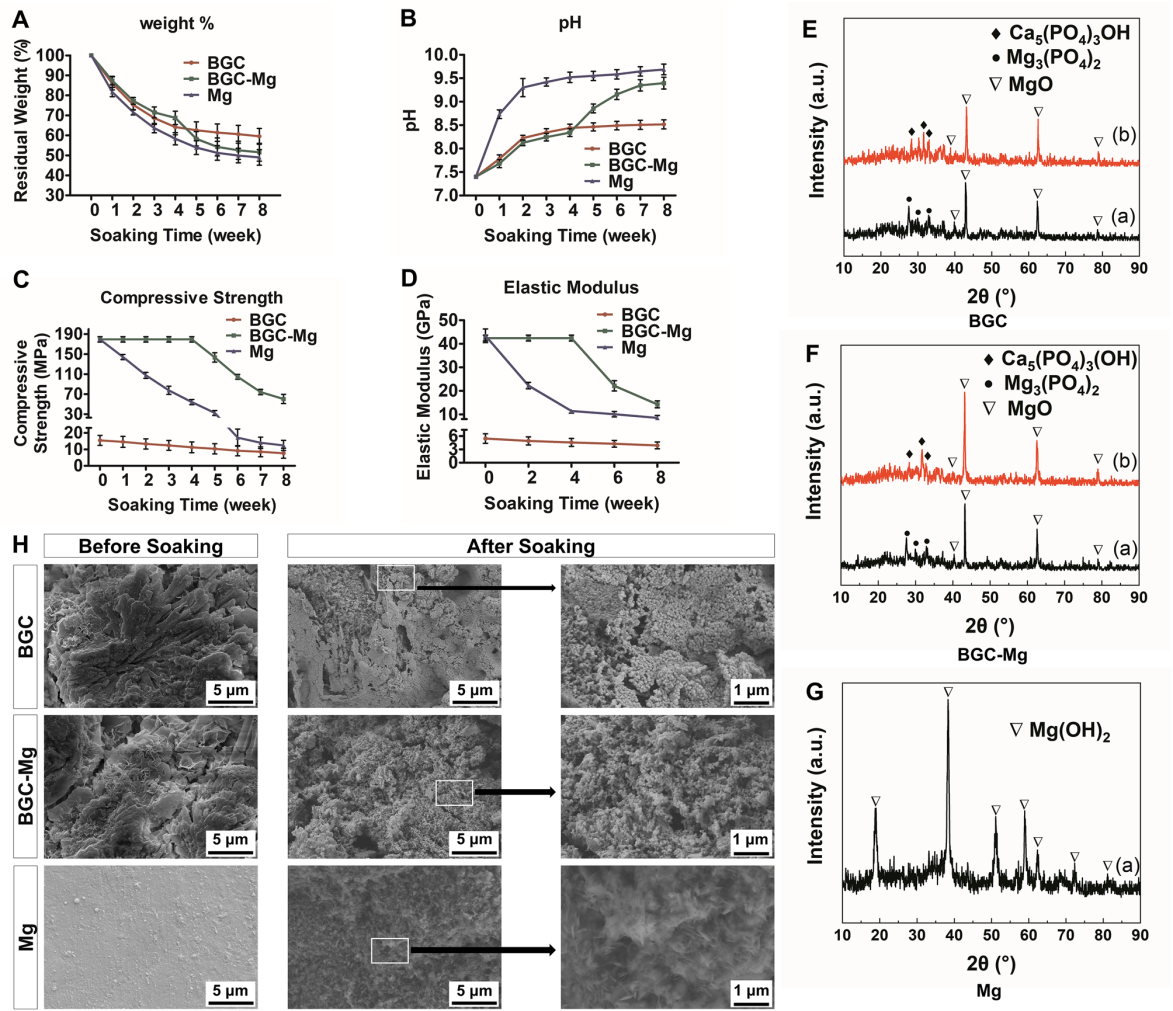
Physicochemical properties of scaffolds during SBF immersion. (**A**) Residual weight of the scaffolds, (**B**) pH of after-soaking SBF of scaffolds, (**C**) Compressive strengths of scaffolds, (**D**) Elastic modulus of scaffolds in different soaking time interval. X-ray diffraction spectrum of BGC scaffold (**E**), BGC-Mg scaffold (**F**) and Mg scaffold (**G**) before and after immersing in SBF for 28 days. (**H**) The SEM images of the scaffolds before and after SBF immersion for 28 days.

In order to investigate the degradation behaviors of different scaffolds, scaffolds of Mg, BGC and BGC-Mg were immersed in SBF at 37 °C for different periods, and the changes in pH, mass, compressive strength and elastic modulus were measured as shown in Fig. 3A–D. Results show that there are significant weight loss for all the scaffolds in the first week, especially for the Mg scaffold. The weight loss of BGC-Mg scaffold is less than that of BGC scaffold. However, after 4 weeks, Mg scaffolds degrade quickly, leading to the quick degradation of the BGC-Mg (Fig. 3A). The pH of the scaffolds immersed solutions were recorded as shown in Fig. 3B. The pH of all samples increased gradually and then reached a dynamic equilibrium in 4 weeks. However, there was a second increase of the pH of BGC-Mg scaffold immersed solution after 4 weeks. The compressive strengths of scaffolds were measured as shown in Fig. 3C. The compressive strength of BGC scaffold was approximately 16.0 MPa, while that of the BGC-Mg composite scaffold was 180.0 MPa, closing to that of cortical bone as100–200MPa^24^. Moreover, the elastic modulus of the composite scaffold was 42.5GPa, which is about twice of 15–25GPa of cortical bone^15^ (Fig. 3D). The compressive strength of BGC-Mg composite scaffold was almost completely retained at 180.0 MPa at beginning 4 weeks, and then decreased to 100 MPa in 6th week, which is still much higher than that of BGC scaffold and can match the normal cortical bone.

The variation of Ca, Mg, Si, B ions concentrations in soak solution were recorded as well (shown in Supplementary Fig. S2). Both BGC scaffold and BGC-Mg scaffold show gradually increase of Mg, Si and B ions, indicating the degradation of scaffolds. The decrease of Ca ions are attributed to the formation of hydroxyapatite, which is confirmed by the XRD of scaffolds after SBF soaking (Fig. 3E,F). The XRD spectrum of Mg scaffold shows sharp characteristic peaks at 2θ = 38.3°, which can be attributed to the reflections of Mg(OH)_2_ in consistence with JCPDS No.86-0441 (Fig. 3G).

The surface morphology of all the scaffolds were observed by SEM before and after soaking in SBF for 8 weeks (Fig. 3H). There were large amount of nanoparticles on the surface of BGC scaffold and BGC-Mg scaffold, referring to XRD spectrum (Fig. 3E,F), these nanoparticles were HA, suggesting that BGC possess high biological activity. While there are rough Mg(OH)_2_ on surface of Mg.

### Biocompatibility of the scaffolds

**Figure 4:**
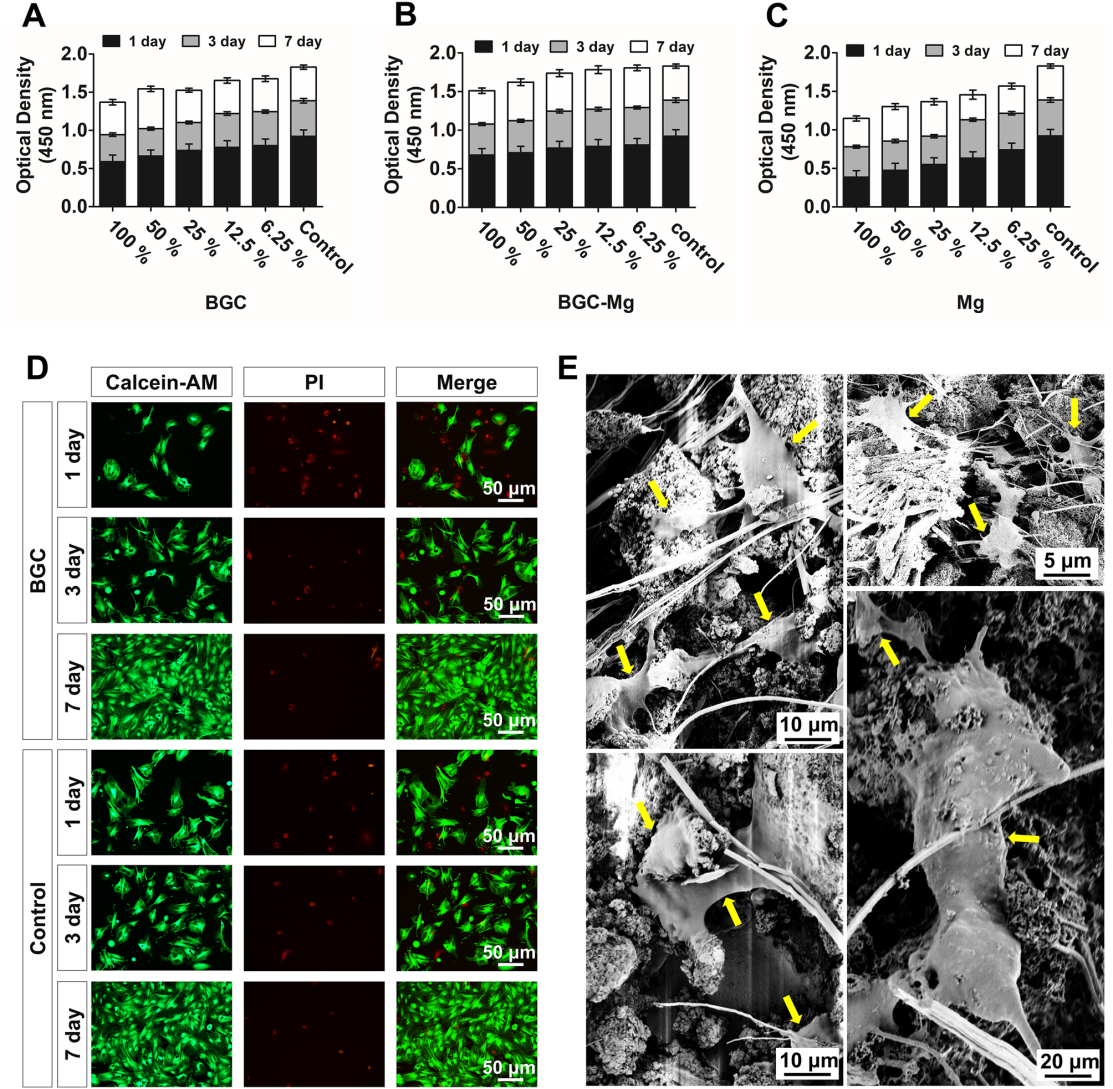
Effects of scaffolds on cell proliferation in vitro. CCK-8 assays of rBMSCs with (**A**) BGC, (**B**) BGC-Mg, (**C**) Mg scaffold respectively at different culture time. (**D**) Fluorescene images of rBMSCs stained with calcein-AM (live cells, green fluorescence) and PI (dead cells, red fluorescence) with scaffolds at different culture time. (**E**) SEM micrographs of rBMSCs proliferated on BGC after 1 d of culture, and yellow arrowheads indicate the rBMSC cells on BGC. Cells in the control groups were cultured in normal medium.

The effects of scaffolds on cell growth were checked using CCK-8 and live-dead cells staining^9^ (Fig. 4). Cell viability assays were firstly carried out using extracts of scaffolds at different concentrations. Compared to the control group (rBMSCs cultured in normal medium), both BGC group and BGC-Mg group showed a slightly decrease of cells viability in high concentrations of extracts (100%), while other conditions no obvious influence on cell growth was found (Fig. 4A,B). However, extracts of Mg group revealed obvious cell growth inhibition at all concentration. The higher concentration of Mg extract, the higher of cytotoxicity was (Fig. 4C). Then the cell viabilities were further assayed by live-dead cells staining using Calcein-AM and PI staining after 1, 3, 7 day of cell culture, where green fluorescence indicated live cells, red fluorescence indicated dead cells (Fig. 4D). No remarkable difference in the number of live cells was found between BGC and control group, which is in correspondence with the results of CCK-8 assay. Furthermore, cell adhesion assay was carried out by seeding rBMSCs onto BGC scaffolds and observed by SEM after co-culturing for 7 d (Fig. 4E). Cells attached tightly on scaffold surface and showed well-flattened and expanded with no significant growth retardation, indicating the BGC shows well biocompatibility with rBMSCs.

### Osteogenic differentiation of rBMSCs in vitro

**Figure 5:**
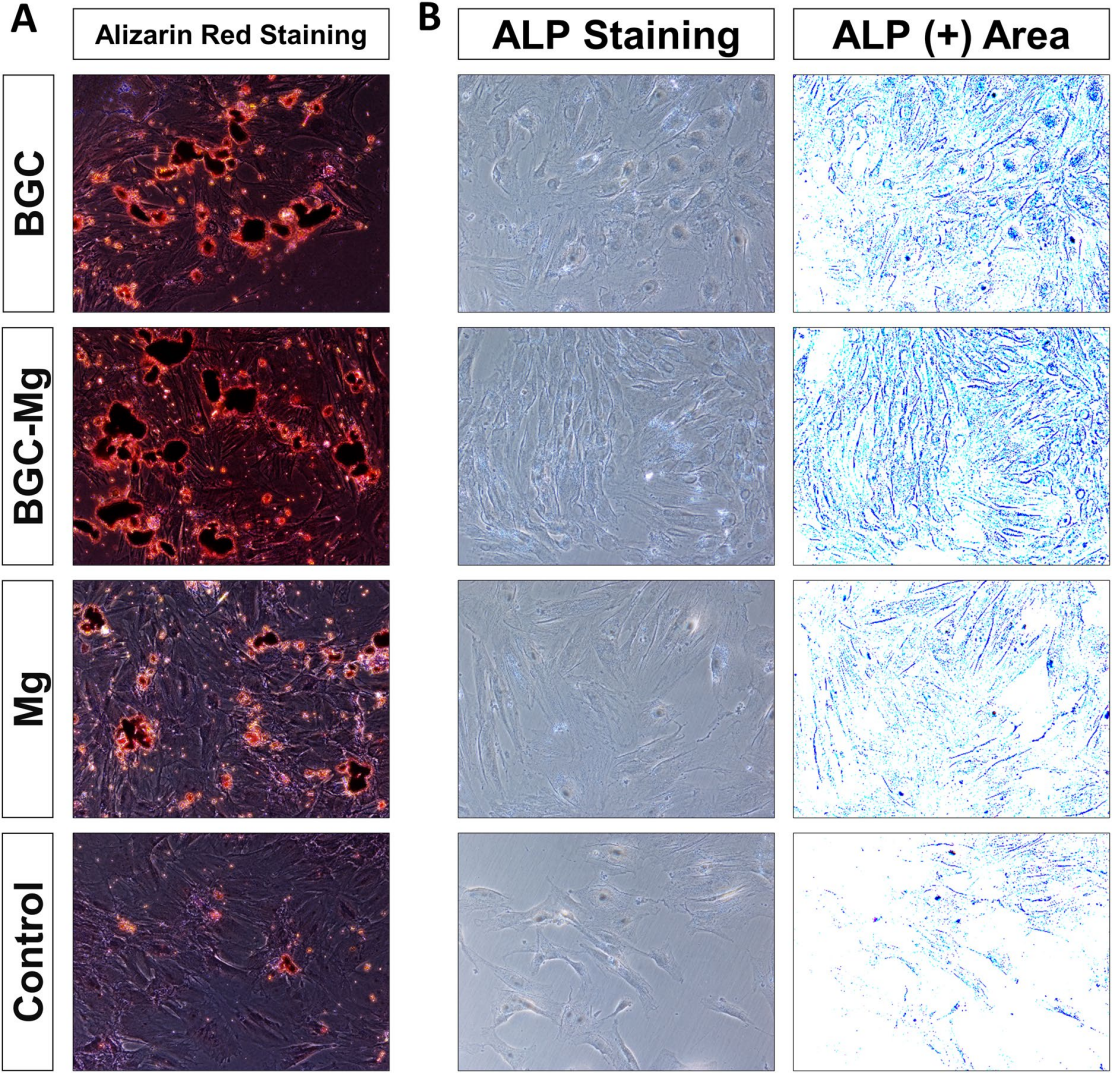
In vitro osteoinduction evaluations of scaffolds for material-guided bone regeneration. (**A**) Optical microscopic images of Alizarin Red S-stained cells at day 21. (**B**) ALP (alkaline phosphatase) staining images of the cells at day 7. The culture medium contained the osteogenic medium.

The differentiation of rBMSCs cultured with the scaffolds was assessed in terms of alizarin red S staining and alkaline phosphate (ALP) activity (Fig. 5)^7^. The results of alizarin red S staining showed that BGC increased mineral deposition of rBMSCs indicating the osteogenic effect of BGC. The ALP activity of rBMSCs with the BGC was much higher than that of the control, indicating the significant increased osteogenic differentiation of the cells.

### X-ray and Micro-CT analyses of bone regeneration by scaffolds in vivo

**Figure 6:**
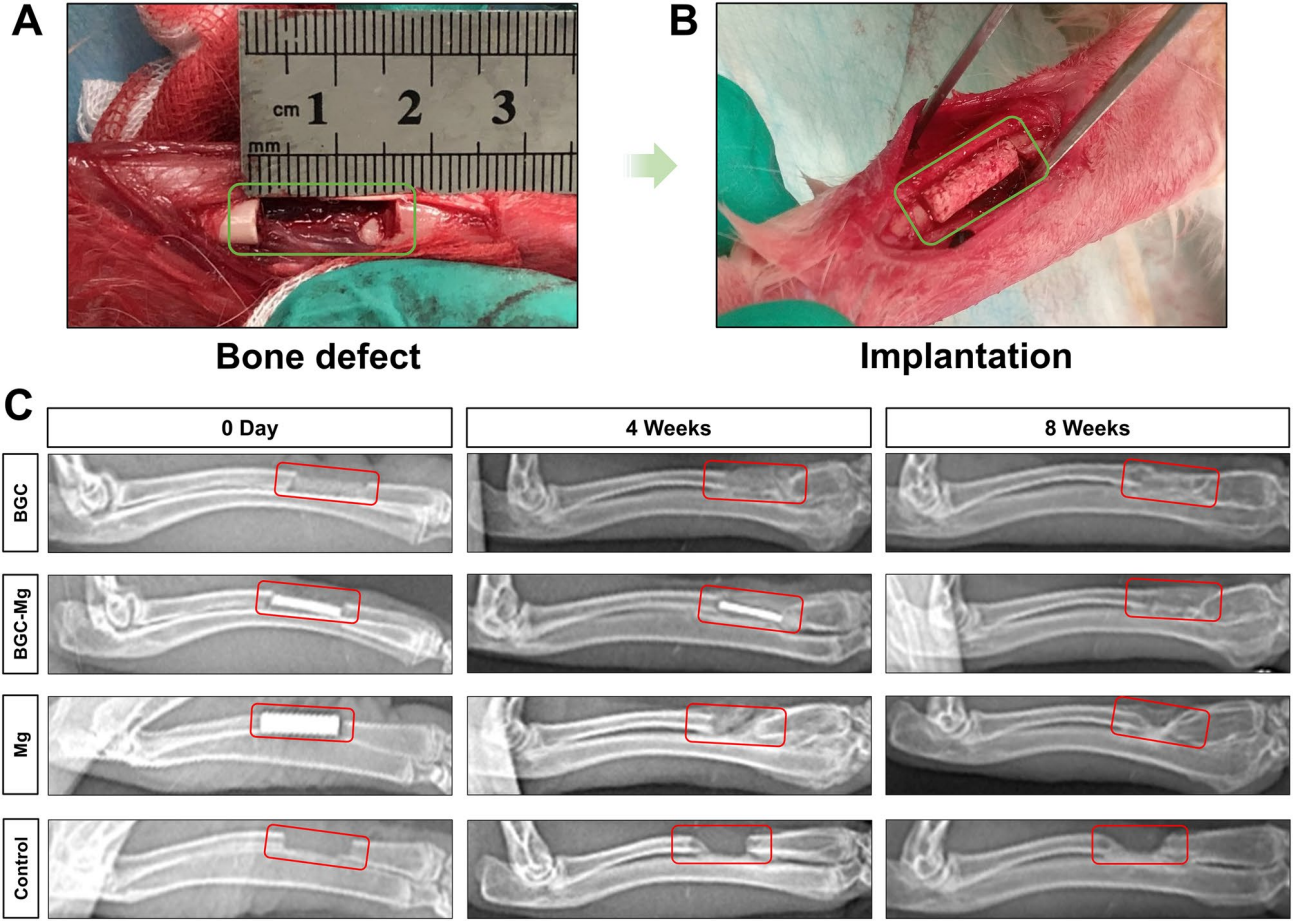
In vivo osteogenesis performance of scaffolds. (**A**,**B**) Surgical procedure for creating bone defect model in rabbits. (**C**) X-ray images of radius regeneration after 0 day, 4 and 8 weeks of surgery. The bone defects in control group were kept empty.

We adopt Radius bone defect of New Zealand white rabbit as the animal experimental model. The defects were implanted with BGC scaffolds, BGC-Mg scaffolds and Mg scaffolds respectively, while defects of blank group were kept empty as control. No signs of infection were observed. We used X-ray to radiograph the rabbits in order to evaluate the degree of scaffolds degradation and bone formation after 4 and 8 weeks of implantation (Fig. 6). In the BGC group, an obvious calcified area was observed around the scaffold after implanting 4 weeks, but the calcified density of bone was lower than that of normal bone tissues. While the bone defect region was filled with bone tissues and completely connected with the host bone margin after 8 weeks. In the BGC-Mg group, Mg alloy was still visible in 4 weeks, indicating well protection of Mg by the PCL coating. But after 8 weeks, Mg alloy disappeared and was replaced by new bone. However, in the Mg alloy group, the Mg alloy scaffolds without PCL coating degraded rapidly in 4 weeks. It is similar with the blank group, the bone defect remained vacant and could not repair itself.

**Figure 7:**
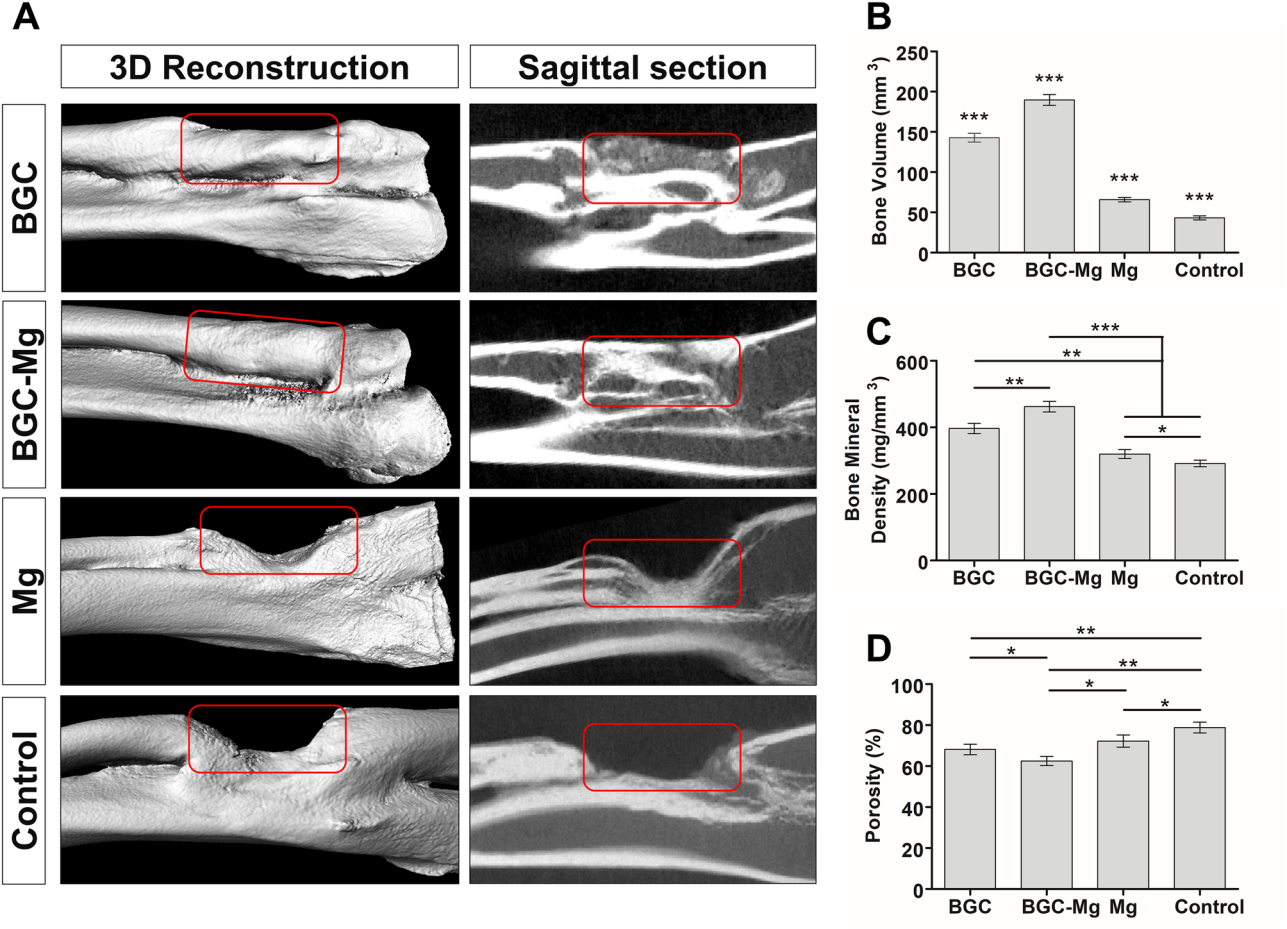
Micro-CT analyses of new bone formation after 8 weeks of surgery. (**A**) micro-CT images of rabbit radius after 8 weeks of surgery. (**B**) Bone volume analysis (***p < 0.001, versus every other group), (**C**) Bone mineral density (*p < 0.05, **p < 0.01, ***p < 0.001 versus the indicated group), (**D**) Porosity of newly-formed bone in the defected areas (*p < 0.05, **p < 0.01, and ***p < 0.001 versus the indicated group). The bone defects in control group were kept empty.

The bone regeneration ability was also studied by microscopic computed tomography (micro-CT) after implantation for 8 weeks (Fig. 7A). Both the BGC and BGC-Mg scaffolds group showed the defect region were almost completely occupied by high density new bone. While minimal new bone formation was observed in the Mg group. There was no evidence of bone defect repair for the blank group. Quantitative analyses of fundamental parameters based on the histomorphometric micro‐CT analysis were presented, such as bone volume (Fig. 7B), bone mineral density (Fig. 7C), porosity (Fig. 7D), and parameters of bone trabecular (Figure S3, Tb. N: Trabecular Number, Tb. Th: Trabecular Thickness, Tb. Sp: Trabecular Separation). All results showed the BGC-Mg scaffolds group present the optimal values.

### Histological analyses of bone regeneration by scaffolds

**Figure 8:**
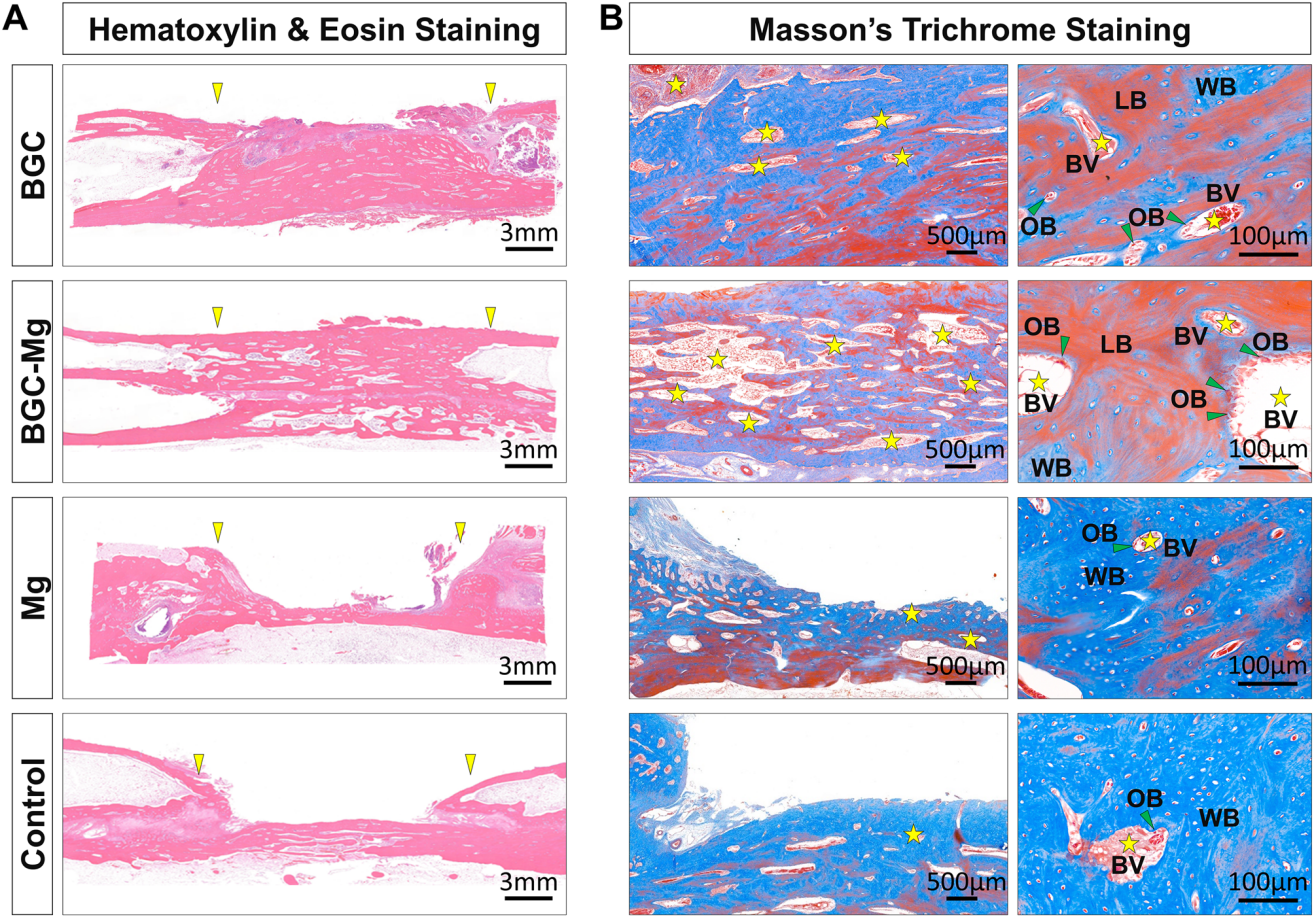
Histomorphometric analyses of bone defect sections after 8 weeks of surgery. (**A**) H&E staining images of the bone defect sections of all groups (The yellow arrowheads indicate the defect site). (**B**) The masson’s trichrome staining images of bone defect sections of all groups (the yellow pentagrams indicate the blood vessels; the green arrowheads indicate the osteoblast cells; LB, lamellar bone; WB, woven bone; BV, blood vessel; OB, osteoblast cell). The bone defects in control group were kept empty.

Masson’s trichrome (MT) and H&E staining were used to evaluate the bone regeneration quality of all groups^8^ (Fig. 8). In H&E-staining, quantities of new bone tissues were clearly observed in the BGC and BGC-Mg group compared to the Mg and blank group, consistent with previous radiographic results. The margins of defect on the BGC and BGC-Mg scaffolds were connected to the host bone for further new bone formation. In the high-resolution images of the MT-staining, a well-arrayed lamellae of bone matrix with quantities of osteoid seams and blood vessels were displayed in the BGC and BGC-Mg group, whereas few newly formed woven bone was found in the Mg and blank group. These results suggested that the BGC-Mg possessed a remarkable osteopromotive ability to facilitate not only quantities of new bone formation but also a high grade of bone maturation in bone defect sites.

**Figure 9:**
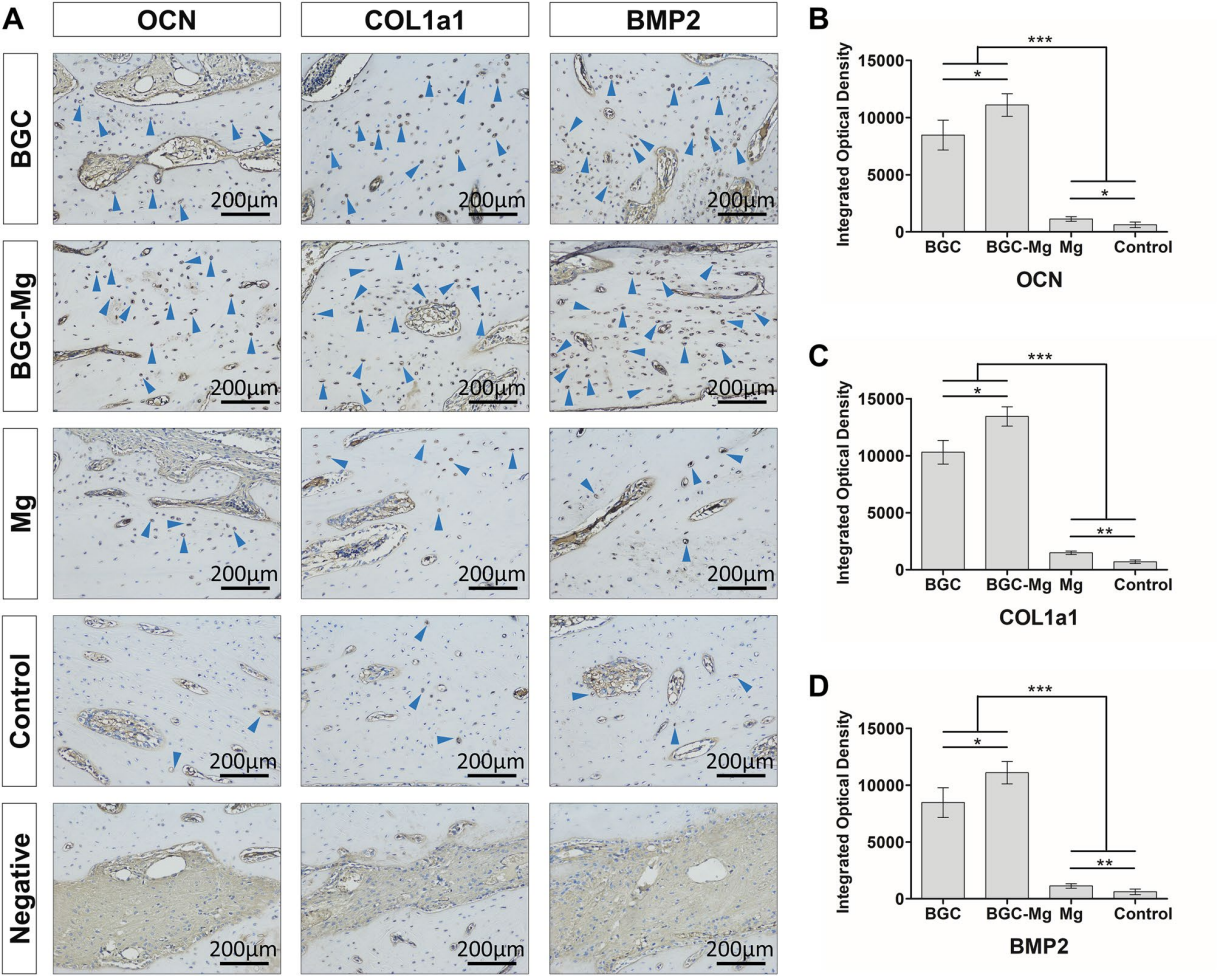
Immunohistochemical analyses. (**A**) Immunohistochemical images of OCN, COL1a1 and BMP2 expressed in newly-formed bone in defected area (blue arrowheads indicate the expression region of each protein). Integrated optical density (IOD) value measured by image analysis of OCN (**B**), COL1a1 (**C**) and BMP2 (**D**) (*p < 0.05, **p < 0.01, and ***p < 0.001 versus the indicated group). The bone defects in control group were kept empty.

To further confirm bone regeneration, immunohistochemistry of bone formation-related proteins were performed (Fig. 9). The level of BMP2 were observed around the newly formed bone of the BGC and BGC-Mg group, implying that the BGC may induce the expression of BMP2, and thus to stimulate osteoblasts to form new bone. Meanwhile, higher level of expression of the COL1a1 and OCN were observed on the BGC and BGC-Mg scaffolds groups (Fig. 9A, blue arrow heads). As shown in Fig. 9B–D, integrated optical density (IOD) values of the BGC and BGC-Mg group were much higher than that of Mg and control group. These results indicated that the BGC and BGC-Mg scaffolds possessed excellent bone formation ability and high efficiency of bone regeneration.

## Discussion

A variety of synthetic bone scaffolds have been developed in the past decades^6,28,29^. However, lack of mechanical property or ostoeconductivity has hampered their wide application^17,25^. In this study, a novel scaffold with cortical bone-matching mechanical properties and ostoeconductivity has been designed inspired by the structure of steel reinforced concrete architectures. In this scaffold, PCL coated magnesium alloy rod resembles the inner steel of reinforced concrete providing excellent mechanical properties, while the bioglass-magnesium phosphate bone cement matrix provides excellent ostoeconductivity. Compressive strength of this scaffold was 180.0 MPa, which is close to that of cortical bone which has range of 100–200 MPa. The similar compressive strengths could avoid the stress shielding between implants and host bone, thus promoting endochondral and intramembranous bone formation^30^. During the degradation of the scaffolds in SBF, hydroxyapatite formed on the BGC surface, suggesting favorable bioactivity of the scaffolds^26,31^.

BMSCs were cultured on the scaffolds and showed proliferation on the scaffolds. Cells viability assayed by CCK-8 and live-dead cells staining showed that the cells viability on the scaffolds was similar to the control group (Cells in the control groups were cultured in normal medium) in 7 days, demonstrating biocompatibility of the scaffolds. Enhanced calcium mineral deposition was detected on the scaffolds by Alizarin red S staining. A significant increase of ALP activity of scaffolds was observed compared to the control group, suggesting ostoeconductivity of the scaffolds.

Bone regeneration of the scaffolds in vivo was carried out using a rabbit radius bone defect model. After 8 weeks of implantation, the defect was significantly filled with newly formed bone both in the BGC and BGC-Mg group, as illustrated in the X-ray and micro-CT images. BGC partially degraded in 4 weeks, and finally was replaced by newly formed bone in 8 weeks, suggesting that BGC and BGC-Mg scaffolds steadily degraded during the bone regeneration process. The ions released by scaffolds may have stimulated the bone formation^8^. The ICP results indicated that Mg, Si, B ions were released from the scaffolds, but Ca ions were deposited on the scaffolds. These results suggest that enhanced bone formation was mediated by bioactive ions from the degradation of BGC and magnesium alloy^14,15,32,33^.

Histological results showed that there was a larger amount of bone formation in the BGC and BGC-Mg group whilst minimum new bone was found in the blank group, which was in agreement with the radiographic results. Masson’s trichrome staining revealed that the newly formed bone by BGC-Mg scaffold was mostly lamellae bone, and the bone mineral density was higher than the other groups (equivalent to 93% of cortical bone). The edges of scaffolds were connected to the host bone for further new bone formation. It is worth noting that an active bone remodeling process was observed in the BGC and BGC-Mg groups. It consists of active hyperplasia of osteonal basic multicellular units, abundant blood vessels, osteoblasts and frequently coupled with osteoclasts. Immunohistochemistry results showed that there were obvious up-regulating expression of osteoblast differentiation marker proteins in the BGC and BGC-Mg group. These results indicated that BGC-Mg scaffold features remarkable in vivo osteogenic capability, promoting new bone formation and subsequent bone maturation within the defected area.

In the aspect of clinical translation, firstly, there have been many studies on the effect of scaffolds porosity, mechanical properties, and degradation on bone defect repair, but no agreement concerning the optimal values, which is very important for future clinical applications. Besides, bone defects are diverse, so personalized therapy becomes popular. The areas and shapes of bone defects in patient are different in clinical practice, but the design of magnesium-alloy rods reinforced bioglass bone cement composite scaffolds hardly meets the requirements of each patient. Fortunately, depending on the computer and 3D printing technology, scaffolds accommodate to different locations, forms, and mechanical requirements may be expected to solve this problem. Finally, the current production technology of magnesium-alloy rods reinforced bioglass bone cement composite scaffolds is still facing many limits, such as small production scale and low efficiency. These problems limited the clinical application of magnesium-alloy rods reinforced bioglass bone cement composite scaffolds and increasing the economic burden on patients. Therefore, it is urgent to simplify the productive process and enhance the output and quality of scaffolds.

## Conclusions

In conclusion, inspired by the structure of the reinforced concrete, we have designed and developed a high-strength scaffold with surface-coated magnesium alloy rod as the load-bearing skeleton and bioglass-magnesium phosphate cement as the osteoconductive matrix. This scaffold possesses cortical bone-matching mechanical properties and excellent osteoconductivity. The strength of the scaffold decreases slowly during biodegradation, while new bone formation matched the degradation of the scaffold. The ions released from the BGC and magnesium alloy may have promoted osteoblast differentiation and up-regulate osteogenic genes and proteins expression, resulting in new bone formation and subsequent bone maturation. This high-strength scaffold has potential in accelerating bone tissue growth in load-bearing cases in the clinic.

## Materials and Methods

### Preparation of Borosilicate bioglass (BG) and bioglass- magnesium phosphate bone cement (BGC)

Preparation of Borosilicate bioglass: 10 g Ca(NO_3_)_2_·4H_2_O (CAS 13477-34-4, Macklin, Shanghai, CHINA) was added into 2 ml of HNO_3_ (0.5 mol/L, CAS 7697-37-2, Macklin, Shanghai, CHINA) solution in the beaker, after thoroughly stirring, 4 ml absolute ethanol (CAS 64-17-5, Sigma-Aldrich, St. Louis, MO, USA) and 10.32 ml of tetraethyl orthosilicate (TEOS, CAS 78-10-4, Sigma-Aldrich, St. Louis, MO, USA) and 755 μL of triethyl phosphate (TEP, CAS 78-40-0, Sigma-Aldrich, St. Louis, MO, USA) were added into the beaker in sequence, then above solution was sealed and stirred for 2 h. After adding 6.19 ml of tributyl borate (TBB, CAS 121-43-7, Sigma-Aldrich, St. Louis, MO, USA), the solution was sealed and stirred for another 2 h, followed by aging at 37 °C for 8 h until the solution transformed into a gel. After drying at 80 °C for 6 h and calcining at 800 °C for 2 h, the resulted calcined BG was carefully ground into fine powder and sieved to about 200 μm for further use^24^. Preparation of bioglass- magnesium phosphate bone cement (BGC): 1.25 g of the above calcined bioglass powder, 2.03 g of calcined MgO (CAS 1309-48-4, Macklin , Shanghai, CHINA) and 1.72 g of KH_2_PO_4_ (CAS 7778-77-0, Aladdin, Shanghai, CHINA) powder were mixed with 3 g of deionized water, then the mixture was poured into a 3D printed mold and pulled out from the mold after 10 min aging, resulting to the BGC^32,34^.

### Preparation and surface modification of magnesium alloy rods and BGC-Mg matrix composite scaffolds

Magnesium alloy rods (diameter = 2 mm, length = 15 mm) were prepared through vacuum melting method in which the proportion of Mg, Zinc, and Ca is 68wt%, 28wt%, 4wt%. For the surface modification of magnesium alloy rods, Polycaprolactone (PCL, molecular weight = 80,000, CAS 24980-41-4, Macklin, Shanghai, CHINA) was firstly added into dichloromethane (CAS 75-09-2, Aladdin, Shanghai, CHINA) with the mass ratio of PCL to dichloromethane is 1:25, then the mixture were heated to 50℃ at a speed of 3℃/min and were stirred till PCL were absolutely dissolved, then the magnesium alloy rods were dipped into the PCL solution and kept for 10 secs before removing from the solution. After holding in air at room temperature for 1 min, the PCL coated magnesium alloy rods were immersed into ethanol for 5 min to extract the remained dichloromethane^35,36^. The above surface modification process was performed once. To prepare the composite scaffolds, 1.25 g of Bioglass powder, 2.03 g of calcinated MgO and 1.72 g of KH_2_PO_4_ powder were mixed with 3 g of deionized water, then the mixture was poured into a prepared molds with the PCL modified magnesium alloy rods in the center (as shown in Supplementary Fig. S1), then the composite scaffold was removed from the mold after 10 min of cement solidification.

### Biodegradation and bioactivity of the scaffolds

The ability of forming Hydroxyapatite (HA) onto the scaffold was measured by immersing in the simulated body fluid (SBF, PHYGENE, Hercynian, Qinghai Province, CHINA), which is a crucial method assessing the in vitro bioactivity of materials^33,37^. BGC scaffold, BGC-Mg scaffold and pure Mg scaffold were immersed in SBF at 37 °C for 1, 2, 3, 4, 5, 6, 7, and 8 weeks, then rinsed thoroughly in acetone (CAS 5000-48-6, Macklin, Shanghai, CHINA) and dried in room temperature for 2d. Weight loss of the scaffolds and pH variation of the fluid were recorded, and the element content of Ca, Mg, Si, B of the after-immersing SBF were measured by inductively coupled plasma atomic emission spectroscopy (ICP-AES). We tested the mechanical properties of the scaffolds according to ISO 6004:2002. The specimen geometry is a cylinder which length is 50 mm and diameter is 10 mm for compressive strength, and a cylinder which length is 10 mm and diameter is 10 mm for elastic modulus.

### Cytocompatibility of the scaffolds

The Cytocompatibility of scaffolds were assessed by cck-8 assays, live-dead cells staining and cells adhesion of scaffolds^38^. Firstly, scaffolds were soaked into cell culture medium for 24 h as a ratio of 3cm^2^/ml according to ISO10993-12:2007, then above mixture was collected as extract concentration and diluted by medium to different concentration (100%, 50%, 25%, 12.5%, 6.25%, 3.125%). The rBMSCs were seeded in 96 well plate (6 × 10^3^/well) and then cultured in different concentrations of the extracts. After 1, 3, and 7 days of culture, cell counting kit-8 (CCK-8, Abcam, Shanghai, CHINA) was added to each well, incubated for 2 h at 37 °C, and cellular metabolic activity was measured by optical density at 450 nm using a microplate reader. For the live-dead cells staining, cells were seeded in 24 well plate (2 × 10^4^/well), then the growth medium in the wells were replaced by extract liquid of the scaffolds(50% concentration), after 1, 3, and 7 days of culture, Live-Dead Cell Staining Kit (Calcein-AM and PI, Abcam, Shanghai, CHINA) was added to each well, incubated for 30 min at 37 °C, then the live cells (green) and dead cells (red) were observed with fluorescence excitation of 490 nm and 535 nm by Fluorescence microscope. For cells adhesion, scaffolds were put in the 24 well plate after sterilization, cells were seeded in 24 well plate with scaffolds (4 × 10^4^/well). After 12 h of co-culture, cells on scaffolds were observed by scanning electron microscope.

### In vitro osteogenic differentiation of scaffolds

The in vitro osteogenic differentiation of scaffolds were assessed by ALP staining and alizarin red S staining. Briefly, rBMSCs were seeded at a density of 2 × 10^4^ cells per well in a 24 well plate for ALP staining, while a density of 1 × 10^5^ cells per well in a 6 well plate for alizarin red S staining^39^. Then scaffolds were placed into wells inoculated with cells for stabilizing overnight, the culture medium (Gibco, Thermo Fisher Scientific Inc. Grand Island, NY,USA) was changed to the osteogenic medium (OSM, comprised of 10 nM of dexamethasone (Dex), 50 mg/mL of ascorbic acid (AA), and 10 mM of b-glycerophosphate (b-gp) in growth medium, Biological Industries, Kibbutz Beit Haemek, Israel). Mineralization was detected by alizarin red S staining after 21 days of culture, cell differentiation was studied by ALP staining after 7 days of culture. Alizarin red S staining (Sigma-Aldrich, St. Louis, MO, USA) was performed according to the manufacturer's instruction. ALP staining (Sigma-Aldrich, St. Louis, MO, USA) of rBMSCs was performed according to the manufacturer's instruction, the stained cells were photographed using a microscope.

### In vivo rabbit radius bone defects repair

Animal experiments were carried out on rabbit radius bone defects model. All animal use procedures were according to the NIH guide for the Care and Use of Laboratory Animals (NIH Publications No. 8023, revised 1978) and were approved by the Experimental Animal Ethics Committee of Nanchang University. Twelve New Zealand white male rabbits, 6 months old with 2.5–3 kg of weight, were randomly divided into four groups corresponding to blank, BGC, BGC-Mg and Mg scaffolds. 3 rabbits were used for each group. All the rabbits were anesthetized with chloral hydrate (10%, v/v, 2.5 ml/kg, CAS 302-17-0, Aladdin, Shanghai, CHINA), and a 20 mm longitudinal incision was made along the radius. After the skin and musculature were separated, a 15 mm bone defect was made using a reciprocating saw. The bone defect models were established and divided into above four groups. Experimental groups BGC and BGC-Mg and Mg represents implanting with BGC scaffolds, BGC-Mg scaffolds and Mg scaffolds respectively, while the blank group was kept empty as control. The incisions were closed using resorbable suture, and the rabbits were given three days of intramuscular injection of penicillin (CAS 69-57-8, 61-33-6, Aladdin, Shanghai, CHINA) 10,000 units per day. The rabbits were sacrificed with an overdose of chloral hydrate and tissue harvest after 8 weeks of surgery^40^.

### Image-based analyses (X-ray and Micro-CT) and histomorphometric analyses

To evaluate new bone formation in the bone defect sites, the surgery regions were radiographed using an X-ray instrument at each time point which indicates the dynamic changes of the scaffolds^41^. Radiographs were obtained at a suitable magnification, and the degree of new bone formation was determined by the grey scale from the X-ray imaging system. For micro-CT observation^42^, the radius was scanned using a micro-CT imaging system with 60 kV and 300 µA. After micro-CT analysis, the harvested bone specimens were fixed in 10% formalin (CAS 50-00-0, Aladdin, Shanghai, CHINA), dehydrated with a graded ethanol series, defatted with chloroform (CAS 71-55-6, Macklin, Shanghai, CHINA), decalcified using 0.5 M EDTA (pH 8.0, Sigma-Aldrich, St. Louis, MO, USA) for 30 days, and embedded in paraffin blocks sequentially. Vertical sections with a 5 µm thickness were cut from the middle of defect using a microtome, and then stained with H&E (Solarbio, Beijing, CHINA) and Masson's trichrome (Solarbio, Beijing, CHINA) for microscope observation^43^. New bone area was measured using the PhotoShop software (Adobe Systems Inc. USA) and calculated by using the following equation: New bone area (%) = An/Ao × 100%, where An and Ao are the new bone area and original defect area, respectively. For this analysis, eight images were randomly obtained in the same section. For immunohistochemistry, the slides were stained with anti-Bmp2, anti-Col1a1 and anti-OCN antibodies (Thermo Fisher Scientific Inc. Grand Island, NY, USA)^44^. Integrated optical density (IOD) value of the positive area of immunohistochemistry images were measured by Image-pro plus 6.0 (Media Cybernetics, Inc, Rockville, MD, USA).

### Statistical analysis

All experiments were repeated a minimum of three times. Experimental results are presented as the mean ± the standard deviation (SD). Data were analyzed by a two-tailed Student's t-test as appropriate for the data set. Statistical analysis was performed using SPSS 19.0 software (IBM Corporation, USA). Values of *p* < 0.05 were considered significant, while *p* < 0.01 were considered very significant.

## Supplementary information

Supplementary Information
