# Supplementary material for: Magnesium-alloy rods reinforced bioglass bone cement composite scaffolds with cortical bone-matching mechanical properties and excellent osteoconductivity for load-bearing bone in vivo regeneration
Source: Sci Rep. 2020 Oct 23;10:18193. doi: 10.1038/s41598-020-75328-7 (PMC7585427; doi:10.1038/s41598-020-75328-7)
Supplement: Supplementary file 1 — Supplementary Information [file 41598_2020_75328_MOESM1_ESM.doc]

**Supplementary material**

Magnesium-alloy rods reinforced bioglass bone cement composite scaffolds with cortical bone-matching mechanical properties and excellent osteoconductivity for load-bearing bone *in vivo* regeneration

Huyang Duan^1,2^, Chuanliang Cao^3^, Xiaolei Wang^4^, Jun Tao^1^, Chen Li^1^, Hongbo Xin^4^, Jing Yang^5*^, Yulin Song^1*^, Fanrong Ai^3,4*^

1 Department of Orthopedic Surgery, The Second Affiliated Hospital of Nanchang University, Nanchang, Jiangxi, 330006, China;

2 Department of Orthopedic Surgery, Yingtan People’s Hospital, Yingtan, Jiangxi, 335000, China;

3 School of Mechanic & Electronic Engineering, Nanchang University, Nanchang, Jiangxi, 330031, China;

4 Institute of Translational Medicine, Nanchang University, Nanchang, Jiangxi, 330031, China.

5 School of Pharmacy, University of Nottingham, NG7 2RD, UK;

**
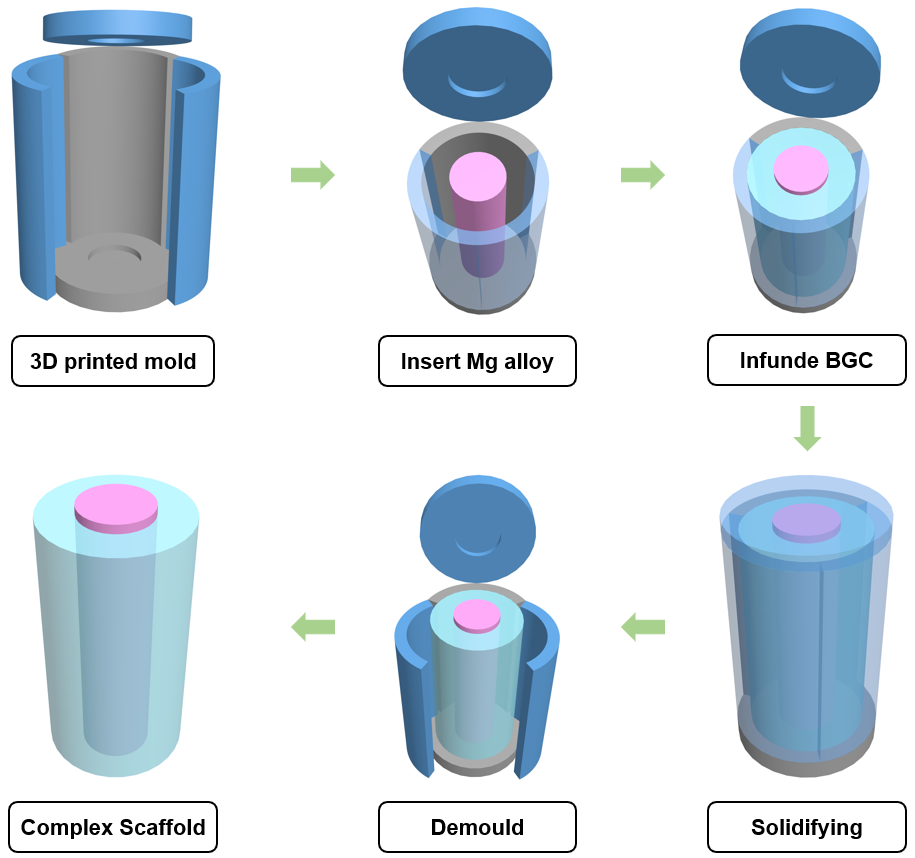
**

**Figure S1.** Design of the 3D-printed mold and preparing process of the composite scaffold.


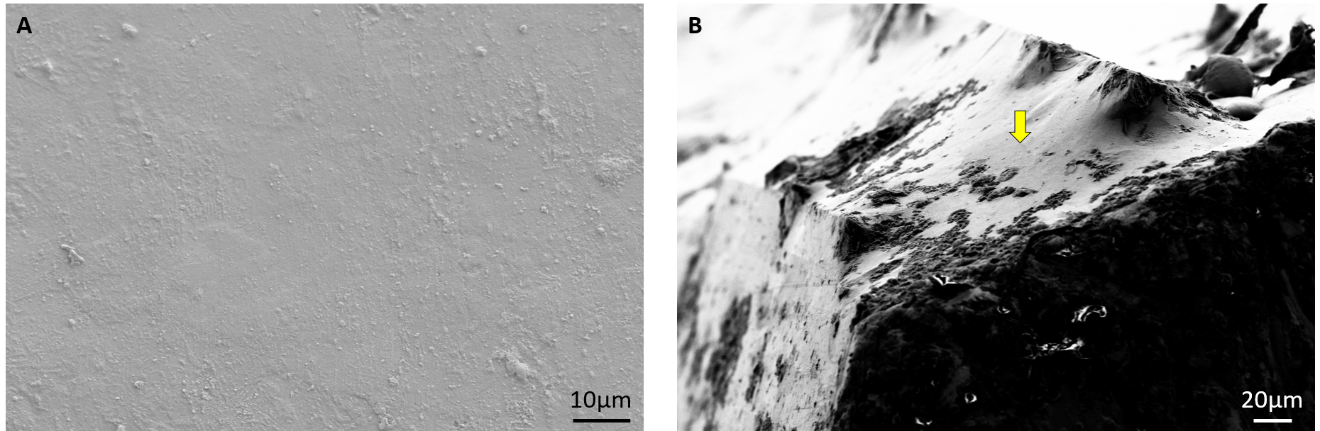


**Figure S2.** SEM images of surface morphologies of Mg alloy (A) and PCL coating (B) (yellow arrowhead indicate the PCL coating).


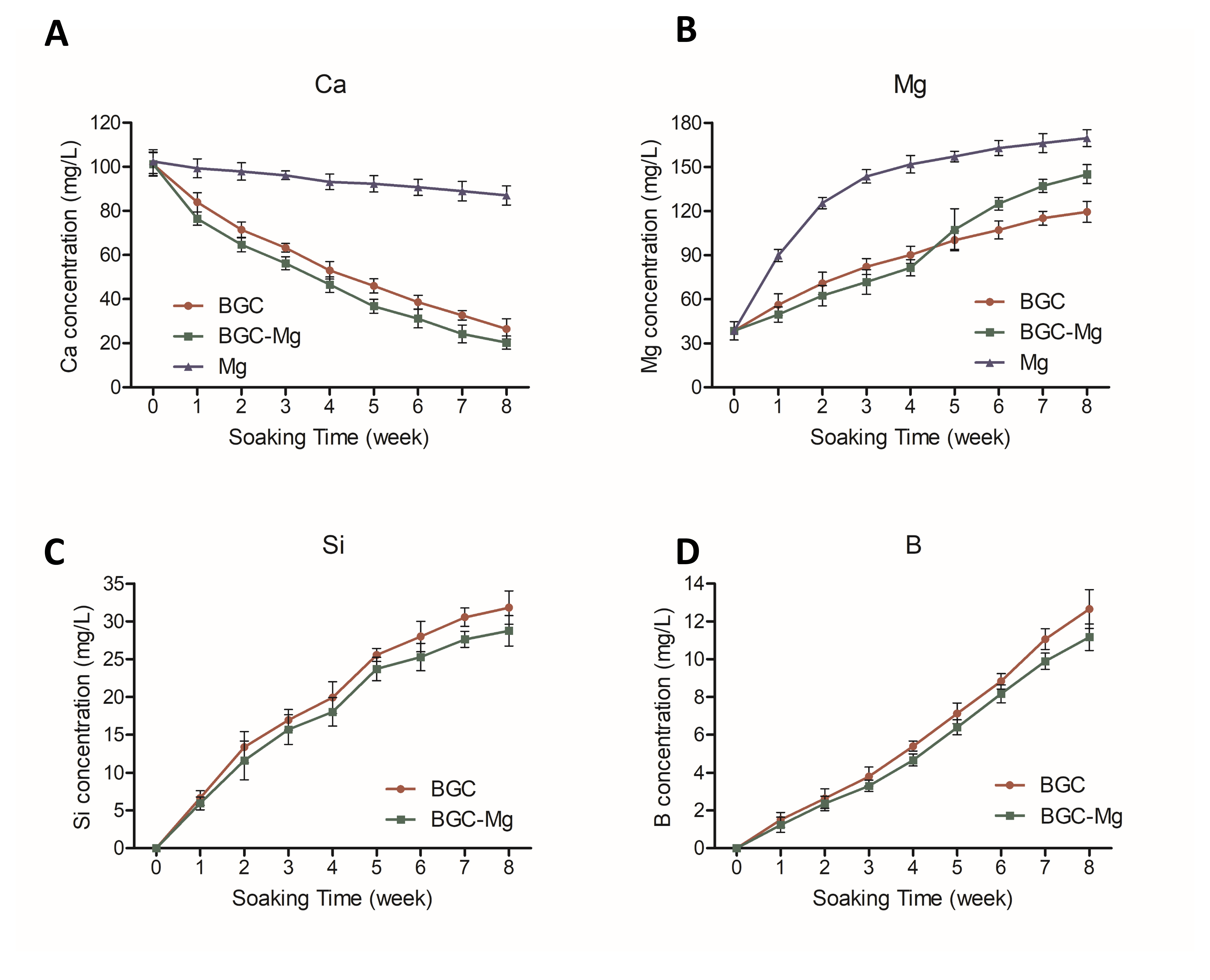


**Figure S3.** Chemical ions concentration in the after-soaking SBF of scaffolds in different soaking time interval. (A) Ca ion, (B) Mg ion, (C) Si ion, (D) B ion.


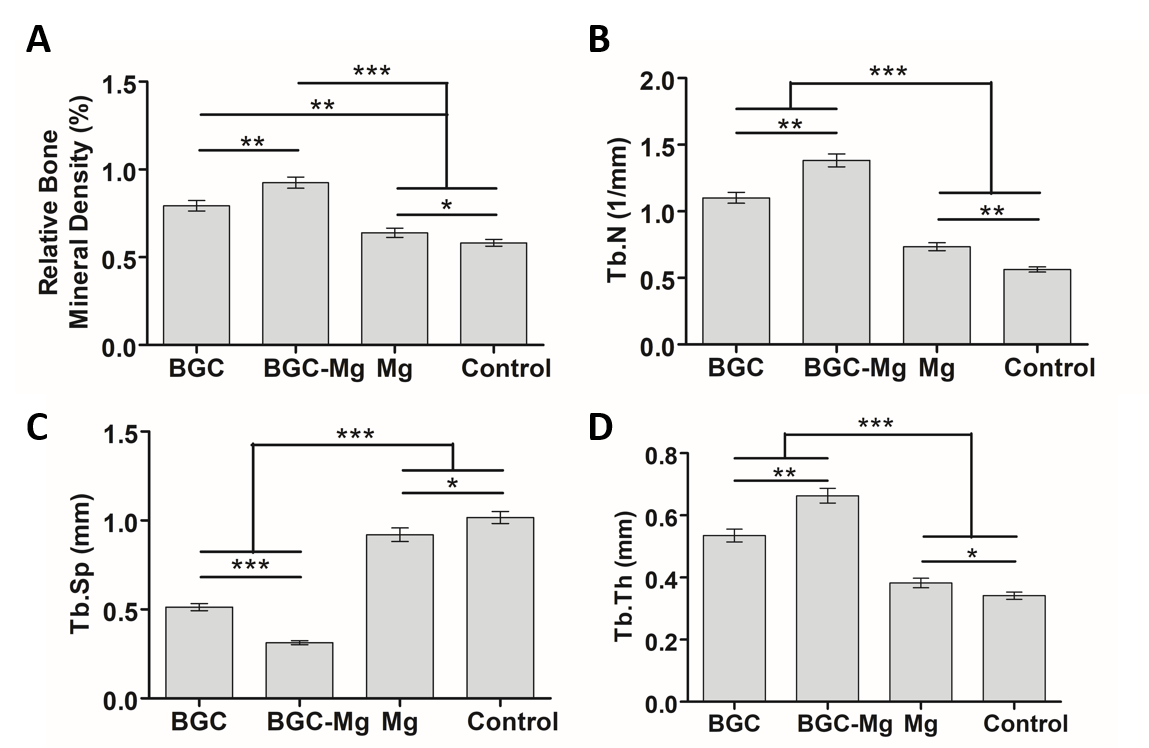


**Figure S4.** Parameter analysis of newly-formed bone based on micro-CT data. (A) Relative bone mineral density of all groups compared with normal bone. (B) The trabecular number (Tb.N), (C) trabecular thickness (Tb.Th), and (D) trabecular separation (Tb.Sp) of the defect site (*p < 0.05, **p < 0.01, ***p < 0.001 versus the indicated group).The bone defects in control group were kept empty.
